# Positive end expiratory pressure and respiratory system resistance between self-inflating bag and T-piece resuscitator in a cadaveric piglet lung model

**DOI:** 10.3389/fped.2022.1014311

**Published:** 2022-11-17

**Authors:** Dharmesh Shah, Mark Tracy, Murray Hinder, Nadia Badawi

**Affiliations:** ^1^ Neonatal Intensive Care Unit, Westmead Hospital, Westmead, NSW, Australia; ^2^ Faculty of Medicine and Health, University of Sydney, Sydney, NSW, Australia; ^3^ Grace Centre for Newborn Care, Children’s Hospital at Westmead, Westmead, NSW, Australia; ^4^ Cerebral Palsy Alliance Research Institute, University of Sydney, Sydney, NSW, Australia

**Keywords:** newborn, resuscitation, T-piece resuscitator, PEEP (positive end-expiratory pressure), respiratory resistance (*R*rs)

## Abstract

**Introduction:**

In neonatal resuscitation, T-piece resuscitator (TPR) are used widely, but the evidence is limited for their use in infants born at term gestation. The aim of this study was to compare the delivered positive end expiratory pressure (PEEP) and respiratory system resistance (*R*rs) using TPR and self-inflating bag (SIB) in a cadaveric piglet model.

**Methods:**

Cadaveric newborn piglets were tracheotomised, intubated (cuffed tube) and leak tested. Static lung compliance was measured. Positive pressure ventilation was applied by TPR and SIB in a randomized sequence with varying, inflations per minute (40, 60 and 80 min) and peak inspiratory pressures (18 and 30 cmH_2_O). PEEP was constant at 5 cmH_2_O. The lungs were washed with saline and static lung compliance was re-measured; ventilation sequences were repeated. Lung inflation data for the respiratory mechanics were measured using a respiratory function monitor and digitally recorded for both pre and post-lung wash inflation sequences. A paired sample *t*-test was used to compare the mean and standard deviation.

**Results:**

The mean difference in PEEP (TPR vs. SIB) was statistically significant at higher inflation rates of 60 and 80 bpm. At normal lung compliance, mean difference was 1.231 (*p* = 0.000) and 2.099 (*p* = 0.000) with PIP of 18 and 30 cmH_2_O respectively. Significantly higher *R*rs were observed when using a TPR with higher inflation rates of 60 and 80 bpm at varying lung compliance.

**Conclusion:**

TPR is associated with significantly higher PEEP in a compliant lung model, which is probably related to the resistance of the TPR circuit. The effect of inadvertent PEEP on lung mechanics and hemodynamics need to be examined in humans. Further studies are needed to assess devices used to provide PEEP (TPR, SIB with PEEP valve, Anaesthetic bag with flow valve) during resuscitation of the newborn.

## What is known?

Use of PEEP is recommended at birth for newborn resuscitation
Neonatal resuscitation guidelines recommend use of T-piece resuscitator to provide PEEP
Inadvertent PEEP increases with increasing lung compliance when using TPR in manikin models

## What is new?

The use of a T-piece resuscitator is associated with higher PEEP in a compliant piglet lung model
Higher inflations rates could lead to inadvertent PEEP and higher respiratory resistance with use of T-piece resuscitator
This study highlights need to assess utility of a T-piece resuscitator in a term newborn resuscitation

## Introduction

The period immediately around birth is a very vulnerable time and the resuscitation skills and equipment available to birth attendants can have a profound impact on the long-term wellbeing of newborns around the world. Use of positive endexpiratory pressure (PEEP) for resuscitation immediately after birth is recommended to improve oxygenation and establish functional residual capacity (1). Neonatal resuscitation guidelines, published by the Australian and New Zealand Committee on Resuscitation (ANZCOR) and the European Resuscitation Council, recommend using a T-piece resuscitator (TPR) to deliver PEEP, rather than a self-inflating bag (SIB) with PEEP valve for term and preterm newborns (2, 3). TPR is widely used around the world, and it works on the principle of a peak pressure-limited fresh flow of gas leaving the TPR circuit to inflate the lung, and an expiratory path with variable resistance to provide adjustable PEEP. This is a similar principle used by Gregory et al. in the treatment of idiopathic respiratory distress syndrome (4).

A review by Roehr et al. examined results from four studies that compared a TPR and SIB during neonatal resuscitation and showed no significant difference in survival at discharge (5). Nor was there a difference in air leak between the two modalities. The studies predominantly looked at preterm infants (<34 weeks gestation) and no clear recommendation for use of PEEP in term infants could be made. The evidence in support of using PEEP in resuscitation of term infants is lacking in human studies although Holte et al. published a randomized controlled trial for resuscitation of term infants, and their findings did not support use of PEEP for resuscitation of term newborns (6).

Drevhammar et al. demonstrated that there was a risk of developing inadvertent PEEP when TPR was used with a higher ventilator rate and shorter expiratory time in a mechanical lung model with a single compartment (7). In a retrospective review of resuscitation of infants of extremely low birth weight using a TPR, Finer et al. reported rapid changes in the PEEP at delivery-suite resuscitation (8). Measured PEEP was as high as 15 cmH_2_O, even though the target PEEP was 5 cmH_2_O. In a single-operator manikin study, Hinder et al. reported a significant increase in inadvertent PEEP with an increase in lung compliance (9). The measured PEEP as a percentage of set PEEP was highest (between 122% and 164%) with lung compliance of 3 ml/cmH_2_O, which is comparable to that of a healthy aerated term newborn lung.

PEEP can vary the airway and endotracheal resistance by varying the time constant. Drevhammar et al. reported increasing the PEEP from 5 to 10 cmH_2_O would increase the time constant from 0.21 to 0.30 s (7). Increasing the gas flow to 15 L/min would significantly reduce the time constant. TPR imposed expiratory resistance of the PEEP valve was shown to increase total system time constant and an increased risk of inadvertent PEEP due to insufficient deflation time. Wald et al. suggested using a higher flow of gas with a TPR during ventilation *via* continuous positive airway pressure to prevent a massive increase in system expiratory resistance (10).

The cardiovascular effects of inadvertent PEEP have been reported in the literature over the last few decades (11). PEEP can lead to increased right atrial pressure leading to a reduced gradient for venous return and cardiac output. It can cause mechanical compression and obstruction of the intrathoracic superior vena cava leading to a fall in cardiac output and systemic hypotension. The adult literature indicates that inadvertent PEEP may be a likely cause of pulseless electrical activity in ventilated patients (12).

This study aimed to determine differences in delivered PEEP and respiratory system resistance (Rrs) in a cadaveric piglet model when using SIB with PEEP valve or TPR when lung compliance was normal or lowered at different peak inspiratory pressures (PIP).

## Methods

### Subjects

Ten freshly euthanized piglets, which had been primarily used for an abdominal study were examined. They were aged 3–5 days, and the mean weight was 1.59 kg. The chest cavity, including the diaphragm, was intact. The abdominal cavity was sutured and intact for this study. They were tracheotomised and intubated with a 4 mm cuffed endotracheal tube. Tracheostomy was performed *via* midline incision on the neck, and the tube was inserted to a depth of up to 5 cm from the skin. Animal ethical approval was obtained from Western Sydney Local Health District Animal Ethics Committee (AEC protocol 5104.06.12).

### Devices

A Laerdal 240 ml SIB with flow diverter and PEEP valve (Laerdal, New York, NY) and Neopuff Infant Resuscitator (Fisher & Paykel Healthcare, Auckland, New Zealand) were used to provide positive pressure ventilation. The SIB, TPR and measurement system were checked for leaks before each data collection session. A calibrated continuous gas flow of 10 L/min was used. A Florian respiratory function monitor (Acutronic Medical Systems, Zurich, Switzerland) or CO_2_SMO respiratory profile monitor (Nova Metrix, Wellingford, CT) was used to measure lung compliance, PEEP and *R*rs. Data from the Florian and CO_2_SMO monitor were collected at 200 Hz *via* an analogue-to-digital converting device, using Spectra Software (Grove Medical) as described in an earlier paper (13).

### Study protocol

**Figure 1:**
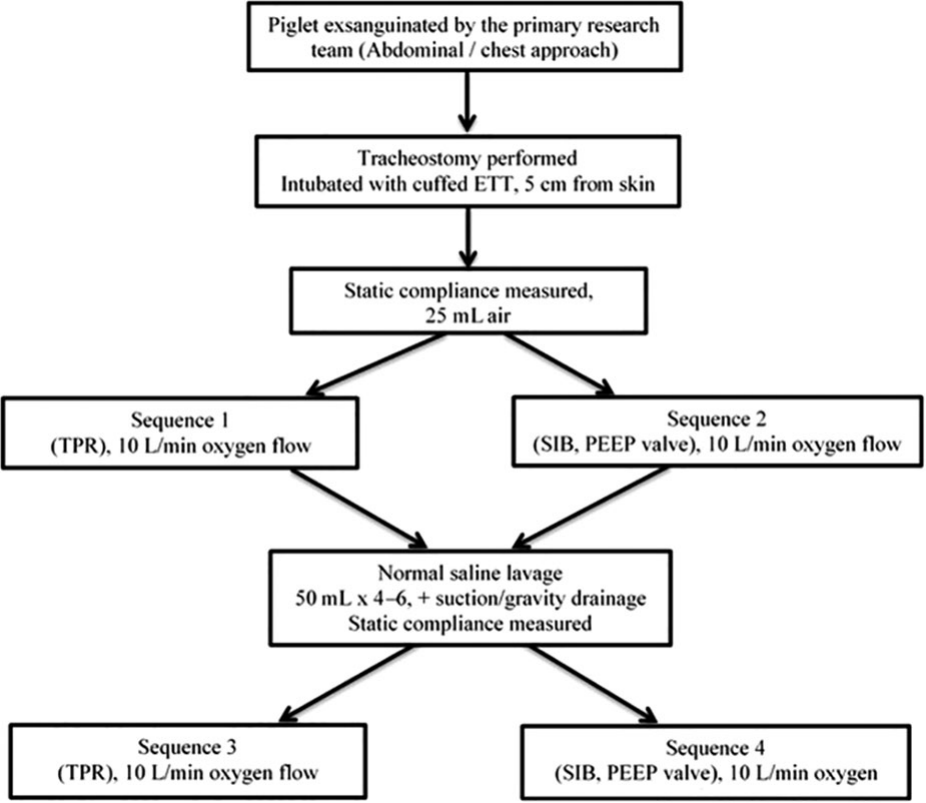
Flow diagram of the study. ETT, endotracheal tube.

The study protocol is presented in Figure 1. Initial static lung compliance (ΔV/ΔP) was measured using 25 ml/kg of gas. Static compliance was calculated using Spectra software (Grove Medical, London, UK). Inflations were given by a TPR and SIB. Variable inflation rates of 40, 60 and 80 inflations per minute (ipm) were used in combination with a PIP of 18 and 30 cmH_2_O. The PIP was measured at patient connection point of each resuscitation device and recorded on the respiratory function monitor. The delivered PIP during PPV with the use of SIB was displayed on the RFM to achieve the targeted PIP. The selection of PIPs of 18 and 30 cmH_2_O was intended to simulate the inflation pressures used for preterm and term neonatal resuscitationas described by Tracy et al. (14). Varying inflation rates were used to simulate breathing pattern of a preterm infant (15). The breaths were timed using a mobile metronome application (metronome by MarketWall.com). Each run was performed with a set PEEP of 5 cmH_2_O for 90 s. A normal saline lavage was performed to wash out the surfactant (25 ml/kg) until the lungs were almost free of surfactant (8–10 cycles) and lung compliance was measured again. The variable rates and PIP were measured again using a set PEEP of 5 cmH_2_O for 90 s each. The circuit was leak free. As shown in Figure 1, there were four sequences. Two occurred prior to the saline wash and two occurred afterwards. In sequences 1 and 2, a TPR and/or SIB was randomly used, and in sequences 3 and 4, a TPR and/or SIB was randomly used.

### Data analysis

Statistical analysis was performed using Stata software (V.13 MP, StataCorp, College Station, TX,). The measured parameters included the mean, minimum and maximum of the PEEP, *R*rs and inspiratory time (Ti). Paired samples *t*-tests were used to compare the means when using a TPR and SIB.

## Results

**Figure 2:**
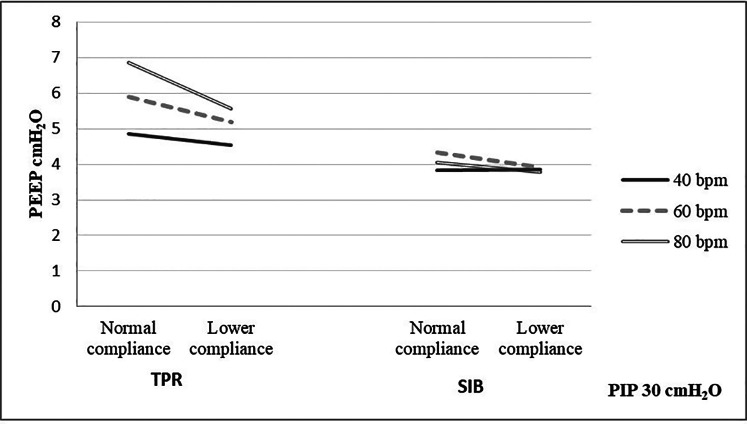
Measured PEEP using TPR and SIB at normal and lower compliance with PIP of 30 cmH_2_O.

**Figure 3:**
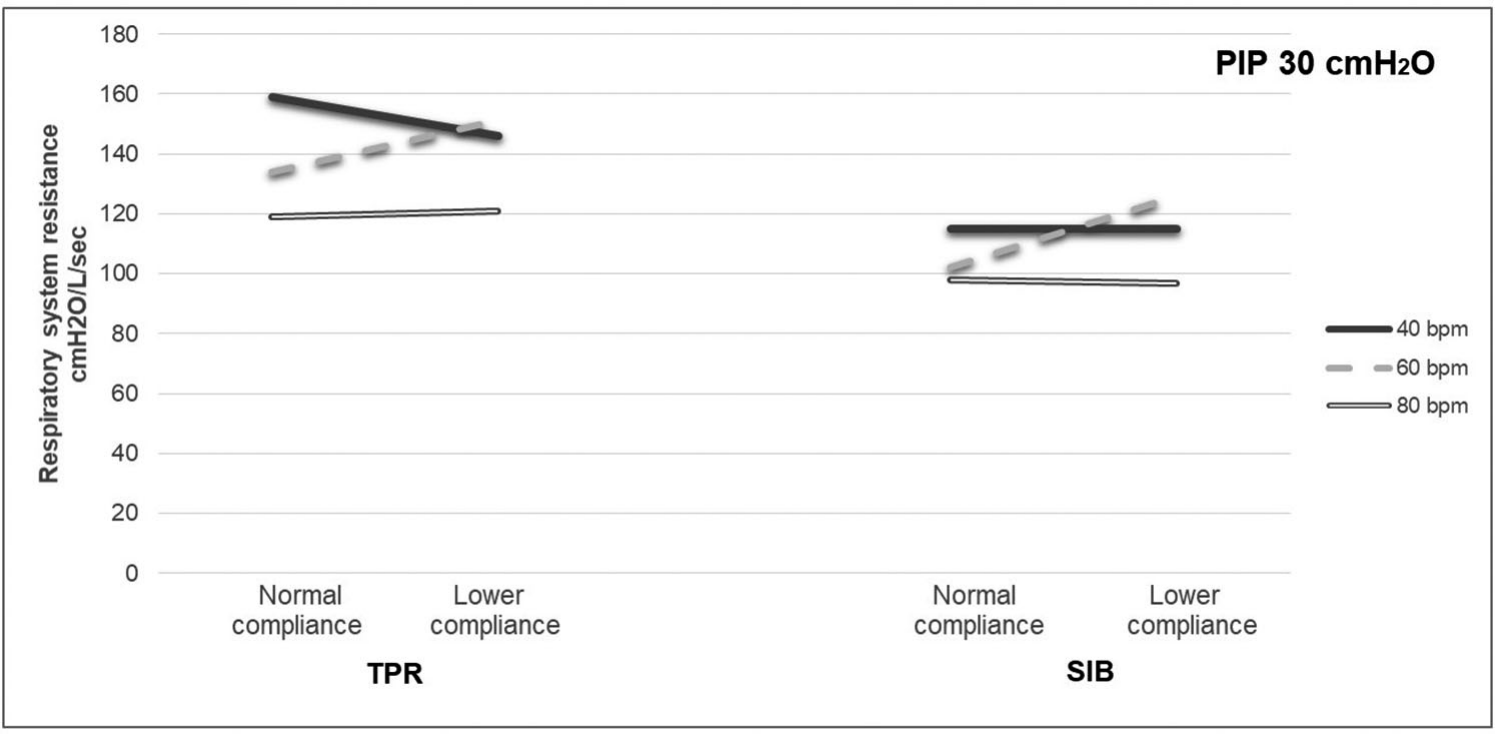
*R*rs using a TPR and SIB at PIP of 18 cmH_2_O.

The mean weight of the piglet was 1.59 kg. The mean initial static compliance was 1.90, and following the saline lavage, it dropped to 1.04. As shown in Table 1, when using a TPR with normal lung compliance and PIP at 18 cmH_2_O, the mean PEEP increased from 4.41 (±0.46) to 5.85 (±0.51) cmH_2_O as the inflation rate increased from 40 to 80 bpm. When using a SIB under the same conditions, the mean PEEP increased from 4.41 (±0.54) to 4.61 (±0.92) cmH_2_O. Similar increases were noted at the PIP of 30 cmH_2_O when using both TPR and SIB with normal lung compliance (Table 1). That is, the mean PEEP increased from 5.10 (±0.50) to 6.94 (±0.79) cmH_2_O as the inflation rate increased from 40 to 80 ipm. When using the SIB, there was a smaller increase in PEEP from 4.37 (±0.98) to 4.84 (±1.16) cmH_2_O, as the inflation rate increased from 40 to 80 ipm. Tables 2, 3 shows the mean *R*rs and Ti at varying inflation rate and Ti using TPR and SIB. Table 4 sets out the mean differences in PEEP and *R*rs when using a TPR and SIB. The mean (TPR vs. SIB) difference in PEEP was statistically significant at higher inflation rates of 60 and 80 ipm when lung compliance was normal (Figure 2). The mean difference was 1.231 (*p* = 0.000) with PIP of 18 cmH_2_O and 2.099 (*p* = 0.000) with PIP of 30 cmH_2_O. At lower compliance, a statistically significant TPR vs. SIB difference in PEEP was observed only at the higher inflation rate of 80 (Table 4). The mean difference was 0.784 (*p* = 0.006) with PIP of 18 cmH_2_O and 1.210 (*p* = 0.003) with PIP of 30 cmH_2_O. The results of paired samples *t*-tests revealed that significantly higher *R*rs was obtained using TPR with higher inflation rates of 40, 60 and 80 ipm and PIP of 30 cmH_2_O (Figure 3), with normal lung compliance (*p* < 0.05, Table 3). There was significantly higher Ti across varying inflation rates and PIP with use of TPR as compared to SIB. The mean difference in Ti (s) between use of TPR and SIB with PIP of 30 cmH_2_O was 0.173, 0.120 and 0.117 (*p* < 0.001) at inflation rates of 40, 60 and 80 bpm respectively.

**Table 1:** Mean (SD) PEEP (cmH_2_O) at PIP of 18 and 30 cmH_2_O with normal and lower lung compliance.

Inflation rate (bpm)	Compliance			
	Normal		Lower	
	TPR	SIB	TPR	SIB
	*M* (*SD*)	*M* (*SD*)	*M* (*SD*)	*M* (*SD*)
PIP at 18 cmH_2_O				
40	4.41 (0.46)	4.41 (0.54)	4.39 (0.29)	3.93 (0.64)
60	5.31 (0.43)	4.52 (0.83)	4.56 (0.62)	4.15 (0.69)
80	5.85 (0.51)	4.61 (0.92)	4.98 (0.48)	4.26 (0.84)
PIP at 30 cmH_2_O				
40	5.10 (0.50)	4.37 (0.98)	4.49 (0.45)	4.28 (0.77)
60	6.07 (0.99)	4.80 (0.65)	5.66 (0.78)	4.72 (1.06)
80	6.94 (0.79)	4.84 (1.16)	6.10 (0.70)	4.89 (1.52)

**Table 2:** Mean (SD) *R*rs (cmH_2_O/L/s) at variable inflation rates and PIP using TPR and SIB.

Inflation rate (bpm)	Compliance			
	Normal		Lower	
	TPR	SIB	TPR	SIB
	*M* (*SD*)	*M* (*SD*)	*M* (*SD*)	*M* (*SD*)
PIP at 18 cmH_2_O				
40	122.8 (58.1)	105.7 (60.2)	128.6 (79.1)	130.3 (83.3)
60	112.0 (51.8)	107.0 (67.7)	124.0 (75.9)	124.8 (59.7)
80	99.7 (48.9)	99.0 (89.2)	145.8 (169.2)	96.2 (42.9)
PIP at 30 cmH_2_O				
40	159.5 (74.6)	115.7 (41.6)	146.3 (85.3)	115.7 (35.9)
60	134.8 (71.0)	102.6 (35.6)	151.6 (120.9)	126.1 (82.0)
80	119.1 (60.4)	98.6 (49.8)	121.3 (77.9)	97.0 (35.3)

**Table 3:** Mean (SD) Ti (s) at variable inflation rates and PIP using TPR and SIB.

Inflation rate (bpm)	Compliance			
	Normal		Lower	
	TPR	SIB	TPR	SIB
	*M* (*SD*)	*M* (*SD*)	*M* (*SD*)	*M* (*SD*)
PIP at 18 cmH_2_O				
40	0.54 (0.10)	0.43 (0.06)	0.47 (0.08)	0.42 (0.08)
60	0.48 (0.02)	0.37 (0.08)	0.43 (0.05)	0.36 (0.03)
80	0.37 (0.02)	0.25 (0.06)	0.34 (0.05)	0.25 (0.03)
PIP at 30 cmH_2_O				
40	0.55 (0.06)	0.39 (0.08)	0.51 (0.06)	0.40 (0.05)
60	0.47 (0.07)	0.35 (0.06)	0.44 (0.05)	0.36 (0.02)
80	0.36 (0.04)	0.24 (0.04)	0.32 (0.04)	0.24 (0.02)

**Table 4:** Mean difference (*M_diff_*) in PEEP (cmH_2_O), *R*rs (cmH_2_O/L/s) and Ti (s) between TPR and SIB.

Inflation rate (bpm)	Compliance			
	Normal		Lower	
	18 cmH_2_O	30 cmH_2_O	18 cmH_2_O	30 cmH_2_O
	*M_diff_* (*p*)	*M_diff_* (*p*)	*M_diff_* (*p*)	*M_diff_* (*p*)
PEEP				
40	0.002 (0.990)	0.727 (**0.039**)	0.464 (**0.041**)	0.210 (0.481)
60	0.792 (**0.004**)	1.266 (**0.002**)	0.411 (0.097)	0.938 (**0.001**)
80	1.231 (**<0.001**)	2.099 (**<0.001**)	0.784 (**0.006**)	1.210 (**0.003**)
*R*rs				
40	17.0 (0.263)	43.7 (**0.015**)	−1.7 (0.484)	30.6 (0.135)
60	4.9 (0.434)	32.2 (**0.027**)	−0.8 (0.491)	25.5 (0.118)
80	0.7 (0.488)	20.5 (**0.035**)	49.6 (0.191)	24.2 (0.091)
Ti				
40	0.140 (**0.002**)	0.173 (**<0.001**)	0.055 (**0.027**)	0.115 (**<0.001**)
60	0.119 (**0.002**)	0.120 (**<0.001**)	0.069 (**<0.001**)	0.087 (**<0.001**)
80	0.118 (**<0.001**)	0.117 (**<0.001**)	0.086 (**0.004**)	0.084 (**0.001**)

**Figure 4:**
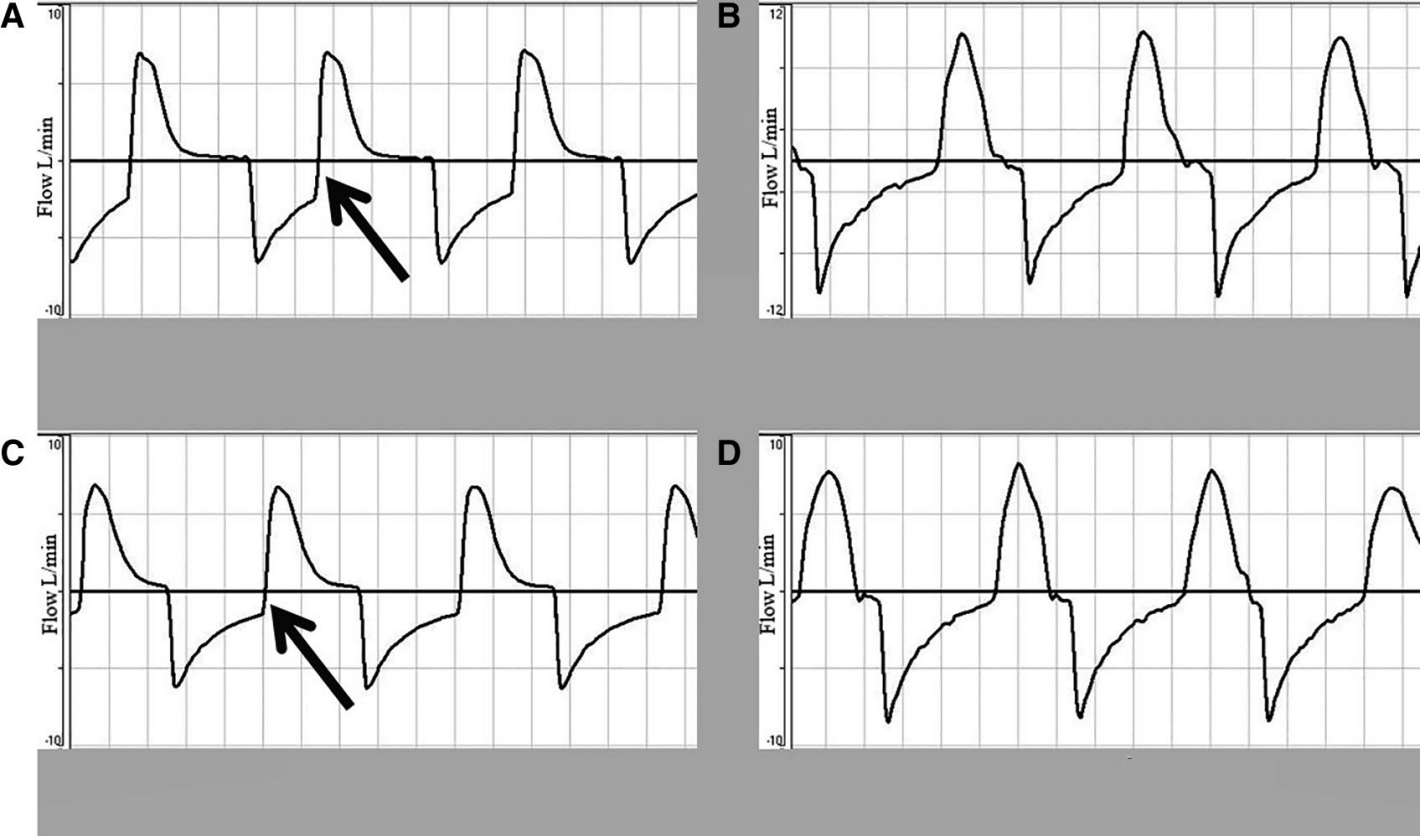
Flow-time waveform showing incomplete exhalation due to rapid cycling to inhalation at PIP of 30 cmH_2_O and 60 inflations per minute. (**A,C**) Depict the flow-time waveform before and after the saline wash using a TPR. (**B,D**) Show the flow-time waveform before and after the saline wash using a SIB.

Figure 4 shows the flow-time waveform during the four sequences. There is incomplete exhalation in the cycles of ventilation with TPR before the saline wash (normal lung compliance), but not when the SIB was used. Breath stacking was observed when using a TPR. In Figures 4A,C, the vertical line at the end of exhalation can be seen in the flow-time curves, which suggests generation of inadvertent PEEP.

## Discussion

This is one of the few studies comparing two ventilator devices and their effect on respiratory mechanics. We conclude that there is a significant increase in delivered PEEP and *R*rs with the use of a TPR in a compliant lung model compared to preterm (lower compliance) lungs. The TPR is widely used for neonatal resuscitation and resuscitation of infants <10 kg around the world and is recommended as the primary modality to deliver newborn resuscitation. In manikin models, it has been demonstrated that a TPR provides consistent and accurate pressures in neonatal resuscitation compared to a SIB (16). However, there is limited evidence for the use of a TPR in infants born at term with normal compliant lungs. The effect of inadvertent PEEP with a TPR in this group of infants is not well studied. Hinder et al. studied TPR on a test lung with varying compliance and reported a significant increase in PEEP with increased compliance (17). Thio et al. examined preterm lambs using a TPR and SIB at set PEEP (5, 7, 10 cmH_2_O) with set PIP of 30 cmH_2_O and varying rates at 20, 40 and 60 ipm with and without gas flow (18). The lung compliance or *R*rs of the system was not recorded. They demonstrated lower mean PEEP in SIB, and in the presence of gas flow with higher inflation rates, resulted in increased delivered PEEP. Our study demonstrated a significant increase in the PEEP and *R*rs with an increase in inflation rate and compliance. Thio et al. used the AMBU Mark IV non-disposable SIB with PEEP valve and the differences in different SIB and PEEP valve needs to be examined. The increase in *R*rs in our study with use of TPR is possibly due to circuit imposed expiratory resistance of the TPR PEEP valve (9). The increase in *R*rs for a fixed compliance and lung volume would results in longer time for the gas to exit the lung and hence increase the risk of breath stacking or inadvertent PEEP. This was aptly demonstrated in our study with significant increase in Ti with TPR compared to SIB leading to lower Te and a possible risk of inadvertent PEEP.

The results reported here are comparable to those of Bennett et al. who reported that use of a SIB with the PEEP valve in place provided significantly less PEEP than both a TPR and flow-inflating bag (SIB: 3.6 cmH_2_O vs. TPR: 4.4 cmH_2_O vs. flow inflating bag: 4.4 cmH_2_O; *p* < 0.005) (19). Transition from a PIP of 20–40 cmH_2_O took significantly longer using a TPR than both a flow-inflating bag and SIB (5.7 s, SD = 2.2 vs. 1.8 s, SD = 0.8 vs. 2.2 s, SD = 1.5, *p* < 0.001). Here, the PEEP was 4.9 cmH_2_O with the use of SIB at inflation rate of 80 bpm and PIP of 30 cmH_2_O, which increased to a mean PEEP of 6.8 cmH_2_O at a similar setting with use of the TPR.

Inadvertent PEEP can be multifactorial. It occurs due to stacking of breaths, which results in early termination of a breath and incomplete exhalation. In a ventilated infant or one who is receiving positive pressure ventilation non-invasively, if there is insufficient expiratory time, this will lead to air trapping and hyperinflation. Hence with higher respiratory rates, insufficient expiratory times and expiratory airflow limitation, the dynamic hyperinflation is exacerbated. Krabbe et al. performed a benchtop study comparing two different TPRs (Neo-Tee vs. Neopuff) at varying flows and set PEEP (20). With PEEP set at 5, 6, 7 and 8 cmH_2_O, the measured PEEP was 5.4, 6.2, 7.2 and 8.6 cmH_2_O respectively. The measured PEEP was significantly higher than claimed by the manufacturer leading to unintended dangerously high pressures. McHale et al. studied operator experience at various ventilator parameters with a TPR (21). Wide variation in mean airway pressure and tidal volume was identified in all groups irrespective of experience.

Diagnosis of inadvertent PEEP is difficult clinically as quite often it is occult. Graphics on a ventilator would help operators interpret the flow-time curves. In a normal ventilation, the expiratory flow (which is a negative deflection on the graphs) will reach zero before the start of the next breath, followed by a period of no flow. In the case of inadvertent PEEP, there is breath stacking, and hence the expiratory flow is cut off prior to reaching zero, initiating the next breath. This generates a vertical line rising from the exhalation part of the flow-time curve (22). Our study demonstrated breath stacking with use of TPR suggestive of inadvertent PEEP. An accidental increase in gas flow during neonatal resuscitation could generate excessive PIP and PEEP (23). There are published reports of the potential hazard of using a TPR in the absence of flow limitation, and researchers have questioned whether the use of a TPR should be restricted to frequent users (23, 24).

Hemodynamic effects of inadvertent PEEP have been well reported in adults, including right ventricular failure and cardiac arrest (22). The effect of PEEP on newborn hemodynamics at resuscitation is not well reported. De Waal et al. studied the effect of PEEP on term and preterm neonates and reported a significant decrease in the superior vena cava diameter and a reduction in right ventricular output (11). Polglase et al. examined effect of PEEP on pulmonary vascular resistance in a preterm sheep model, with an increase in PEEP from 4 to 8 cmH_2_O reducing the pulmonary blood flow by 20.5% (25). We reported an increase in mean PEEP with use of TPR from 4.41 cmH_2_O with a PIP of 18 cmH_2_O and inflation rate of 40 bpm to 6.94 cmH_2_O with a PIP of 30 cmH_2_O and inflation rate of 80 bpm. Our study could not establish the hemodynamic effects of inadvertent PEEP, but users need to be cautioned about the use of excessive and/or inadvertent PEEP.

### Strengths

This is an animal study and hence simulates a complex lung model compared to a manikin model. We were able to compare various respiratory parameters at varying compliance by performing the saline wash out.

### Limitations

The compliance of the piglet lung prior to the wash was 1.9 which is lower than the equivalent compliance of a healthy newborn infant lung at birth. But there was significant drop in the compliance post saline wash. The piglets were day 3 to day 5 of life and hence would not represent the lung dynamics of transition at birth as the lungs have dried and ductus would have closed. The abdominal cavity was opened for another research but was closed for our research, but that could have affected the effect of abdominal pressure on the diaphragm. Higher inflation rates of 80 bpm were used to study the effects on PEEP, although they are not recommended by international resuscitation guidelines (Current guidelines recommend inflation rates of 40–60 bpm and PIP of 20–25 cmH_2_O). The hemodynamic effects of PEEP on pulmonary vasculature could not be studied as the piglets were demised. This was an intubated leak free model and hence effect of mask leak and airways obstruction could not be studied. Only a single type of commercially available TPR was tested in our study.

## Conclusion

The use of a TPR is associated with significantly higher PEEP in a compliant lung model and higher inflation pressure, which is probably related to the resistance of the circuit. Significantly increased Ti with TPR compared to SIB especially at higher inflation rates could lead to inadvertent PEEP. The effect of high and inadvertent PEEP on lung mechanics and hemodynamics needs to be examined. Further studies are needed to assess devices used to provide PEEP (TPR, SIB with PEEP valve, Anaesthetic bag with flow valve) during resuscitation of newborns and infants <10 kg.

## Data Availability

The raw data supporting the conclusions of this article will be made available by the authors, without undue reservation.
